# Supplementary material for: The Fit After Baby randomized controlled trial: An mHealth postpartum lifestyle intervention for women with elevated cardiometabolic risk
Source: PLoS One. 2024 Jan 9;19(1):e0296244. doi: 10.1371/journal.pone.0296244 (PMC10775990; doi:10.1371/journal.pone.0296244)
Supplement: S1 File — (DOCX) [file pone.0296244.s002.docx]

**Detailed Protocol**

**Randomized controlled trial of a mobile health intervention to increase postpartum weight loss in women at increased risk for cardiometabolic disease**

**Principal Investigator: Jacinda M. Nicklas, MD, MPH, MA**

1. **SPECIFIC AIM**

Conduct a randomized controlled trial to compare the Fit after Baby program with a mobile texting control group (Text4Baby).

1. **HYPOTHESIS**

Women randomized to the Fit After Baby program will demonstrate greater weight loss and less postpartum weight retention than women randomized to the Text4Baby group.

1. **BACKGROUND AND SIGNIFICANCE**

**A. Pregnancy as a “stress test”:**

Pregnancy complications provide an early warning of future cardiometabolic risk.^[[1]](#endnote-1)^ This harbinger of risk can identify women long before traditional risk factors for cardiovascular disease and diabetes are detected in the primary care setting.^[[2]](#endnote-2)^ Women with pregnancies complicated by gestational diabetes mellitus (GDM) have a ~50% increased risk for developing type 2 diabetes mellitus within 10 years, are likely to develop atherosclerosis earlier,^[[3]](#endnote-3)^ and have increased risk for hypertension^[[4]](#endnote-4)^ and cardiovascular disease (CVD).^[[5]](#endnote-5),^^[[6]](#endnote-6)^ Preeclampsia, preterm delivery, delivery of a small-for-gestational age (SGA) neonate, hypertensive disorders in pregnancy, and GDM are independently associated with a 50-300% increased risk for CVD.^[[7]](#endnote-7)^ About 30% of US women will have at least one of these predictive conditions during pregnancy.^[[8]](#endnote-8)^

**B. Cardiovascular disease and diabetes confer significant mortality in US women**: Cardiovascular disease is the leading cause of death, and diabetes the seventh leading cause of death, among US women. Among women 45-54, the first decade following the childbearing years, CVD and diabetes are the second and seventh leading causes of death, respectively.^[[9]](#endnote-9)^ Given this early risk, in 2011 the American Heart Association recommended taking a history of pregnancy complications when evaluating cardiovascular risk, and included a history of preeclampsia, gestational hypertension, and/or GDM as major risk factors for CVD.^[[10]](#endnote-10)^ The risk factors revealed in pregnancy are exacerbated by overweight, which increases the risk for CVD and diabetes in women at every age and in every ethnic group, by 40% for overweight and by as much as 300-400% for severe obesity.^[[11]](#endnote-11)^ It is currently estimated that 50-60% of U.S. women of childbearing age are overweight or obese.^[[12]](#endnote-12)^ The cost of obesity in the US is considerable, as much as $147 billion annually.^[[13]](#endnote-13)^ Notably, the presence of obesity among women age 45-54 increases the lifetime cost of cardiovascular disease and diabetes by nearly 200%.^[[14]](#endnote-14)^

**C. The postpartum period is a critical window of opportunity**: Studies demonstrate that pregnancy weight retained beyond 6-12 months postpartum is usually retained long-term and is a powerful independent risk factor for future obesity.^16^ Importantly, up to 60% of previously normal weight pregnant women become overweight in pregnancy and the American College of Obstetricians and Gynecologists considers overweight and obesity the leading cause of maternal and neonatal morbidity.^[[15]](#endnote-15),^^[[16]](#endnote-16)^ Given the significance of postpartum weight retention, the postpartum year is considered a critical window of opportunity to make lifestyle changes to decrease future risk of obesity and chronic disease.^14,^^[[17]](#endnote-17),^^[[18]](#endnote-18)^ Lifestyle changes, including weight loss, smoking cessation, improved diet, and physical activity have been shown to decrease risk for diabetes and CVD.^[[19]](#endnote-19),^^[[20]](#endnote-20),^^[[21]](#endnote-21),^^[[22]](#endnote-22),^^[[23]](#endnote-23)^ Reducing postpartum weight retention also decreases risk for weight-related complications in future pregnancies, including GDM, preeclampsia, and perinatal mortality.^[[24]](#endnote-24),^^[[25]](#endnote-25)^ Lifestyle changes made by mothers in the postpartum period have the potential to improve health of other members of the household, including partners and children, thereby broadening the impact. Studies demonstrate that postpartum women may be particularly receptive to making lifestyle changes given their new awareness of risk factors that were unmasked during pregnancy as well as their motivation to create a healthy home for their offspring.^[[26]](#endnote-26)^

**D. A lack of available treatment options:** Despite the importance and critical timing of the postpartum period, there are currently no clinically available evidence-based programs designed for high-risk postpartum women.^[[27]](#endnote-27)^ Few lifestyle intervention studies have been conducted in postpartum women at elevated cardiometabolic risk.^[[28]](#endnote-28)^ Although the Diabetes Prevention Program (DPP) demonstrated successful weight loss and reduction in diabetes incidence in an older population of pre-diabetics,^34^ women in the DPP with a history of GDM lost less weight and were less likely to sustain changes in physical activity than women without a history of GDM.^[[29]](#endnote-29)^ Studies attempting intensive face-to-face methodologies similar to the DPP in postpartum women demonstrate limited efficacy and poor retention.^[[30]](#endnote-30),^^[[31]](#endnote-31),^^[[32]](#endnote-32)^ This is due, at least in part, to multiple barriers to face-to-face participation described by postpartum women, including time constraints, infant and breastfeeding demands, older childcare responsibilities, and reluctance to spend time away from family.^[[33]](#endnote-33),^^[[34]](#endnote-34)^ Given the high stakes for failure in the face of these barriers, there is increasing interest in using technology to improve the efficacy of lifestyle interventions for this high risk population.^33,43^

**3.1 Innovation:**

**A. Target population:** Despite increasing recognition that obstetric history may identify at-risk women before their physiologic markers or cardiovascular risk score may prompt a referral to a specialist, there are no initiatives in primary care that I am aware of to intervene in this high-risk population. Although a few small-scale time-intensive specialty clinics do exist for postpartum women who have had pregnancy complications,^[[35]](#endnote-35)^ feasible, cost-effective, scalable solutions to address cardiometabolic risk at the population level are sorely needed. Given the current high rates of obesity, CVD, diabetes, and prediabetes (recently estimated to be 38% of US adults),^[[36]](#endnote-36)^ this is a timely and potentially transformative approach.

**B. Use of mHealth:** Women of childbearing age are one of the fastest growing user groups for cell phones and smartphones, across race and socioeconomic class.^20^ Leveraging mobile technology for health promotion provides the unprecedented opportunity to communicate and provide support for behavioral choices in “ecological moments;” real-time moments when people make lifestyle decisions. In addition, mHealth technology greatly facilitates tracking of behavior and weight, allowing for real time recording, feedback, and accountability. In light of escalating health care costs and rapid increases in the incidence of cardiometabolic disease, extending the reach of health promotion into daily life is an innovative approach for high-risk women with multiple and intensive family/work demands. An mHealth lifestyle intervention for the population of women at elevated cardiometabolic risk would leverage the widespread adoption of mobile devices among women of reproductive age, and offer the potential of a scalable and cost-effective program that could extend health promotion beyond the traditional clinic visit into home and daily life.

**C. Evidence-based approach integrating behavioral theory:** Although there has been an explosion of use of mobile phones and health-related apps, the vast majority of available apps do not adequately include evidence-based strategies^[[37]](#endnote-37),^^[[38]](#endnote-38)^ or use behavioral theory,^[[39]](#endnote-39),^^[[40]](#endnote-40)^ and almost none have been rigorously tested.^[[41]](#endnote-41)^ To my knowledge, there are no apps specifically designed for postpartum women at elevated cardiometabolic risk. The majority of postpartum apps available commercially are centered around babies, not mothers’ health, and include activities like tracking infant feedings and sleep. I have developed the mHealth *Fit After Baby* program to address this gap. We have developed the app using evidence-based strategies for weight loss, cardiometabolic disease prevention, and behavior change.

**5. METHODS**

**5.1: Rationale:**

This pilot RCT is designed to provide preliminary data to support the design of a larger pragmatic trial for a future R01 proposal to assess weight loss and impact on risk factors in postpartum women at elevated risk for cardiometabolic disease.

**5.2: Source of subjects and recruitment methods:**

The Colorado study staff has completed training in the protection of human research participants (CITI) and will recruit subjects from the University of Colorado and Denver Health patient populations.

At the University of Colorado Hospital, women who are attending clinic visits at the OB/Gyn clinic will be prescreened via medical records and via our existing treatment relationship. All potential women will be approached for recruitment. Women will be consented during their clinic visit in a private exam room by a study physician or by a professional research assistant (PRA). In addition, women who are inpatients at the University of Colorado will be identified through the recruitment mechanism via the CCTSI. They will be consented in private by a CCTSI perinatal nurse or by the study PRA. Women who are within 12 weeks after a delivery can also be referred to us and enrolled in the study. We will post paper flyers advertising the study near OB clinics and we will advertise on the University of Colorado website for clinical trials and the Anschutz Health and Wellness Center website. Women being seen at clinics outside of UCH may have access to flyers about the study, which would encourage them to call the research assistant for more information.

**5.3 Screening and Informed Consent**

Our in-person/telephone screening questions will address the specific inclusion/exclusion criteria for the study. During this screening period, we will only gather information that is directly related to assessing eligibility and suitability for this study. At the beginning of the screening conversation, we will inform potential subjects of the nature and sensitivity of the questions, ask whether this is an appropriate time for them to answer these questions, and tell them this process is expected to take up to 10 minutes. In the interest of confidentiality, we will only record the subject's initials at the beginning of the screening conversation. Then, we will explain to the subject that she will be asked a set of questions to determine eligibility and that at the end, if she appears to be eligible and is interested in pursuing the study, or if she agrees to have her information filed to enable contact for future studies, she will be asked to provide contact/identifying information.

Informed consent will be obtained by one of the physician study staff, the PRA at University of Colorado, or a nurse from the perinatal CCTSI team. During the first two weeks of recruitment, the PRA will be supervised by senior study staff. Subjects who are being consented by non-MDs will routinely be offered the option to speak with one of the physician study staff with any remaining questions or concerns about the study. Although there are non-MDs who may be obtaining consent, physician study staff will be available by pager if needed during the consent process. All subjects will be at least 18 years old and able to provide consent. We will closely follow the guidelines of the COMIRB during interactions with subjects and during procedures for obtaining informed consent. The consent form will be reviewed in detail with each subject and all questions will be answered. Subjects will be given as much time as they want to read, review, and complete the consent form. Subjects who prefer contemplating their decision for days or up to 6 weeks or who wish to discuss their participation in the study with others (family members, friends, and physician) will be encouraged to do so. We will stress that participation in our research study is voluntary, that subjects may withdraw from the study at any time, and that the investigators reserve the right to discontinue the research protocol at any time. A copy of the signed informed consent form will be given to each subject.

**5.4: Eligibility:**

Inclusion Criteria:

1. Age 18-45
2. Postpartum Body Mass Index (BMI): 26-45 kg/m^2^ (≥24 for Asians)

3) Positive history of one or more of the following complications in most recent singleton pregnancy:

- 1. Gestational diabetes mellitus (by Carpenter-Coustan criteria, IADPSG criteria, or a documented clinical diagnosis). Women with a glucose value >200 mg/dL after a 50-g glucose challenge test at >12 weeks gestation will also be included.
  2. Preeclampsia (high blood pressure and proteinuria diagnosed after 20 weeks gestation)
  3. Gestational hypertension (new hypertension diagnosed after 20 weeks without proteinuria)
  4. Pre-term delivery (32-37 weeks)
  5. Small for gestational age (<10th percentile for gestational age)

4) Access to and be willing to use a wi-fi enabled iPhone (5 or higher) or iPod (5 or higher).

5) Capable of providing informed consent

6) Between 4 weeks and 16 weeks after delivery

Exclusion criteria include:

1. Personal history of Type 1 or 2 diabetes
2. Personal history of breast cancer or any other type of cancer other than a basal cell skin cancer;
3. Personal history of major chronic illness all to be assessed by the study physician for ability to participate, including cardiovascular disease (coronary artery disease, congestive heart failure, valvular heart disease, stroke, transient ischemic attack, or intermittent claudication), kidney disease affecting kidney function severe enough to affect participation, liver disease affecting liver function severely enough to affect participation, venous or arterial thromboembolic disease, untreated adrenal insufficiency, depression requiring hospitalization within the past 6 months, or non-pregnancy related illness requiring overnight hospitalization in the past 6 months;
4. Underlying disease/treatment that might interfere with participation in/completion of the study (e.g. significant gastrointestinal conditions, major psychiatric disorders affecting the ability to participate, and others at the discretion of the study clinician);
5. Re-current pregnancy;
6. Pregnancy of twins or multiples;
7. Diagnosis of diseases associated with glucose metabolism;
8. Current or planned participation in a commercial weight loss program (i.e. Jenny Craig) over the duration of the study;
9. Previous or planned bariatric surgery;
10. Taking certain prescription medications including long-term high dose glucocorticoids, atypical antipsychotics associated with weight gain (such as respirdal (respiradone), clozapine (klozaril), olanzapine (zyprexa), quetiapine (seroquel), etc.)) or weight loss medications including prescription (Qsymia, phentermine, topiramate, Belviq, Contrave, Saxenda, Orlistat), or non-prescription (Alli) medications;
11. Taking metformin or other medications known to affect glucose metabolism during the postpartum period;
12. Other active medical problems detected by examination or laboratory testing, at the discretion of the physician;
13. During the first trimester, any fasting blood glucose > 126 mg/dl, any HbA1c at or above 6.5%, or any plasma glucose >200 mg/dl.

**5.5 Treatment assignment and randomization:**

At their first study visit (approximately 6 weeks postpartum), subjects will be randomized into either:

1. Text4Baby Group: 3 study visits (6 weeks, 6 months, 12 months) OR

2. Fit After Baby mHealth Intervention Group: 3 study visits (6 weeks, 6 months, 12 months) AND interaction with the Fit After Baby mHealth program and lifestyle coach

We will randomize in a 2:1 ratio favoring the intervention arm so that we can collect the maximal amount of data from users of the mobile application. This is important because we are collecting data about use of the mobile application in addition to the primary outcome for efficacy. Having more data on actual users will allow us to be more competitive for a future R01 submission emphasizing dissemination and implementation. We have improved the power of the study by adding subjects. Improving the power of the study will increase our likelihood of finding a true difference, which will in turn improve our ability to be competitive for a future R01 application. We will use a computer program to randomize subjects that will generate random treatment assignments using a permuted block scheme with randomly varying block sizes. We will schedule subjects as closely as possible to the 6 week, 6 month, 12 month dates. However, given potential scheduling conflicts with subjects at the clinics, we will allow a window of flexibility of 4-16 weeks postpartum for the 6-week visit. We will also allow one month before and after the 6-month and 12-month visits. After the trial, we will conduct focus groups with participants (2-4 focus groups, 5-8 per group) to assess barriers and facilitators to adoption and engagement of the Fit After Baby program.

**5.6 Study Procedures:**

In this randomized controlled study participants will be randomly assigned to either a control or an intervention group in a 1:2 ratio. While only the intervention group will have access to mHealth Fit After Baby program and personalized coaching from Lifestyle Coach during the 12-month study period, both control and intervention groups will receive the following:

1. Three assessment visits, conducted at the UCH CTRC. These visits will include the criteria and procedures listed in Table 3 below.

2. Study visit reminder calls 1 week before baseline, 6 months postpartum, 12 months postpartum.

3. A $200 stipend for completing all study visits. This stipend will help cover childcare and transportation costs. The payment is structured as follows: $50 following completion of the 6 week & 6 month visits, $100 following completion of the 12 month visit. Participants will also be given a small gift (<$5 value) at delivery (baby socks) from the study staff for participating in the study, and receive small gifts (<$5) (picture frame or book) by at the 6 month visit and by mail at 9 months, to remind patients about the study. Participants in the Fit After Baby group will have the opportunity to earn $5, $10, $15, and $20 gift cards (total up to $50) as an incentive to complete tasks within the mobile application.

During the COVID-19 pandemic, a COVID-19 lifestyle questionnaire, Edinburgh post-partum depression scale, and pregnancy physical activity questionnaire will be sent via email to all participants to assess the effect of social distancing on physical and mental health. Participants with and Edinburgh score >11 and/or suicidal ideation will be contacted by the PI for follow-up. This questionnaire will take 45 minutes to complete and will be completed using RedCap. It will be sent to participants in May, 2020. A reminder to complete the questionnaire will be sent 2 times, if they have not completed it within 1 week. Participants who complete the questionnaire will be compensated with a $20 Target Gift Card.

a. Study visits and parameters to be measured

Medical Record Review/Diagnosis Confirmation

Upon signing the consent form and enrolling in this research study, all women will be scheduled for their first of 3 study visits approximately 6 weeks postpartum. The consent form will include language to allow study staff access to the medical record information to confirm medical diagnoses by the inclusion criteria stated above. Subjects who do not have medical conditions as defined by the inclusion criteria stated above documented in their medical records will not be eligible to continue in the study. If a subject becomes pregnant during the course of the study, she will be asked to be followed during the pregnancy to determine occurrence of pregnancy complications and gestational weight gain.

Study Visits

At study visits, all women will be assessed for clinical outcomes, behavioral outcomes, mediating variables, and moderating variables (see table 3). Clinically relevant labs (pregnancy tests, OGTT, fasting glucose, HbA1c, TSH, LDL, triglycerides) will be reviewed when results are available. All subjects will complete approximately 45 minutes of questionnaires at study visits.

In accordance with ADA recommendations and UCH standard of care guidelines for postpartum diabetes screening for patients with prior GDM, which suggest 6 week postpartum glycemic status check via oral glucose tolerance test (OGTT), all enrolled subjects with a history of GDM will be offered an OGTT. Study staff will schedule this visit and with the subject’s permission, share OGTT results with subject’s pregnancy care provider (MD or CNM), thereby eliminating the scheduling of duplicate lab appointments. We anticipate this collaboration with OB caregivers will be welcomed, as published data demonstrate clinicians have a difficult time getting these women in for their 6 week postpartum OGTT – less than 50% of women had a 6 week postpartum OGTT.^[[42]](#endnote-42)^

All subjects will be instructed to arrive fasting to study visits, i.e. nothing except water for 10 hours prior to study visit. At study visits up to 50 cc of blood will be drawn, including fasting bloods and optional blood for storage.

During the first study visit, all enrolled subjects will be randomized using previously described permuted block scheme to either control or intervention arm.

Control arm: Subjects who enter the control arm will be shown how to sign up for the free Text4Baby program. Women assigned to the Text4Baby control arm will receive 2-4 free text messages per week from the Text4Baby program,^[[43]](#endnote-43)^ a nonprofit maternal child health program which provides information including baby care and resources for women tailored to their number of weeks postpartum. Since Text4Baby does not emphasize weight loss it will serve as an active control. If women are already enrolled they will be asked to continue to receive texts from the program.

Fit After Baby arm: Women assigned to the Fit After Baby program will use the app (see Table 1 for description of app components), as well as communicate with a lifestyle coach by email or phone. They will receive daily content for 12 weeks, and then less intensive interactions once per week plus home page tracking options until 12 months postpartum. Subjects randomized to the Fit After Baby arm will be shown how to download the app onto their phone. They will also be given a Fitbit and a body weight scale at the 6 week/baseline study visit and shown how to use these.

**Components:**

1) *Fit After Baby* mobile application: The *Fit After Baby* application is an evidence-based lifestyle intervention mobile app currently available for wireless devices running the iOS platform. The *Fit After Baby* program is adapted from the Diabetes Prevention Program^[[44]](#endnote-44)^ and tailored for this postpartum population at elevated cardiometabolic risk and for delivery via mobile app. The 12-week program contains weekly themes:

1) Introduction to the *Fit After Baby* program,

2) Self-monitoring and Energy Balance

3) Physical Activity

4) Stress Management

5) Carbohydrates

6) Fat

7) Protein

8) Portion Control when Eating Out and Eating In

9) Your FAB Environment

10) Staying on Track

11) Keep Up Your Physical Activity

12) Staying Motivated

Each day of the week contains one or more pages of content, and other components include goal-setting, quizzes, and games. There is a mechanism to communicate with the lifestyle coach by email or by text. Once participants receive their Fitbits, their physical activity data will be passively transmitted through wireless/Bluetooth connections (steps, distance, sleep, and calories burned). Participants will manually input their weights and set other goals using the app. They will use the free Fitbit application, linked to the *Fit After Baby* app, to track diet and exercise. Through the *Fit After Baby* application, users will receive real-time feedback on progress towards their weight loss, diet, and physical activity goals. If a participant elects not to do tracking via Fitbit, they will still be able to use the Fit After Baby application. Data from passive (manually inputted) and active tracking will be exported to datasheets and analyzed to assess participants’ adherence to the program, including manually inputted weights, days with physical activity data, transmitted by wearing Fitbit (provided) and/or logging physical activity, and days with dietary intake logged. Given that the app includes an option to manually enter physical activity, the use of Fitbit is not essential to the overall operation of the app and the program.

2) Lifestyle CoachA registered dietitian (RD) with training in patient-centered counseling techniques will work with the participants to help them set and achieve goals. The lifestyle coach will provide feedback, advice, and support through the *Fit After Baby* app, and via text messages and email messages. She will prepare to answer questions related to content for each week, how to use the app, and be available to answer any other questions the participant may want answered.

A study physician trained in patient centered counseling will periodically review email and text interactions, as well as notes from phone calls, to ensure adherence to these techniques. The coach and study physician will view progress of participants through a dedicated portal via an android coaching app where they can track diet, physical activity, weight, and progress towards individualized goals.

After a subject has completed the 12 month visit, we will send results of fasting glucose, lipid profile, hsCRP, HbA1c, and TSH from all study visits. The results will be accompanied by a letter from the study physician.

For women who completed their 6-week visit but do not complete their 6 or 12 month visits, we will attempt to reach them by phone, text, email, and/or mail. Additionally, during the consent process, we will get permission from subjects to contact a family member or friend if we are having trouble getting in touch with the subject at any point in the study.

Study Outcomes:

The primary outcomes are change in weight from baseline to 6 months and to 1 year postpartum (exit study visit), and change in weight from self-reported pre-pregnancy weight to one year postpartum.

Secondary outcomes**:** We will assess engagement and satisfaction with the program as potential mediators, defined as use of the app, adherence to self-monitoring of weight, diet, and physical activity, and interactions with the lifestyle coach. To assess changes in cardiometabolic risk factors we will obtain: blood pressure, waist circumference, and biomarkers at baseline, 6 months, and 12 months. We recognize that some of these measurements will be elevated in the postpartum state, however we hypothesize that we will see a greater change among women randomized to the intervention program. We will also assess sleep adequacy and appetite using scales embedded in the app and also sleep data from the Fitbit.

Recruitment targets and power calculation: We plan to recruit at least 7 participants per month, with a goal of consenting 140 subjects to account for recruitment yield of 50-70%. After 2 years we should have data from 81 participants at one year postpartum. With 54 subjects in the intervention group and 27 subjects in the control group, a 2-sample t-test controlling for baseline weight will have at least 87% power to detect a 4.2 kg difference between groups in 12 month weight change (the 4.2 kg difference with a pooled standard deviation of 5.7 kg was observed in the Balance after Baby RCT). This is adequate power for a pilot study designed to establish the foundation for a subsequent larger pragmatic trial of the *Fit After Baby* program.

+Structured interviews

**3rd trimester**

**Delivery**

**Recruitment**

**4 wks**

**12 weeks**

Baseline visit

mHealth *Fit After Baby* program

Text4Baby

**Randomization**

**Study visit measures: weight, height, blood pressure, waist circumference, glucose, HbA1c, insulin, adiponectin, lipids, hsCRP, physical activity, depression, hCG, optional stool sample**

**Flow of *Fit After Baby* RCT**

**12 months**

Final visit

**6 months**

6 month visit

**Measures:** Body weight and height: We will determine weight, measured twice wearing light clothing. We will obtain pre-pregnancy weight by self-report. Others have shown a high correlation (R=0.99) between self-reported and clinically measured pre-pregnancy weight.^[[45]](#endnote-45)^ We will measure height and use kg/m^2^ to determine BMI. 2) Demographics: We will collect age, self-reported ethnicity and race, working status, parity, family history of diabetes and cardiovascular disease, prior personal history of pregnancy complications, education level, marital status, number/ages of children living at home, and number of other household members. 3) Pregnancy test: We will obtain urine for rapid determination of hCG at the follow-up visit at 6 and 12 months postpartum. If the test is positive the participant will be referred to her clinician for confirmatory testing. We will not do hCG tests at the baseline visit, since many women will still have positive levels from pregnancy. If a woman is determined to be pregnant during the study year they will be censored at that timepoint. We do not anticipate any harm to participation even if a woman were to become pregnant during the study. 4) Waist Circumference: Per NHANES methodology.^[[46]](#endnote-46)^ 5) Blood pressure: Determined twice, five minutes apart in the seated position after 15 minutes of rest. Values will be averaged. 6) Biomarkers: HbA1c, hsCRP, adiponectin and fasting glucose, insulin, and lipids. 7) Potential mediators: Diet will be assessed by 2005 Block FFQ, administered online during the study visit. Physical activity will be assessed using an adapted Pregnancy Physical Activity Questionnaire (PPAQ).^[[47]](#endnote-47)^ Breastfeeding intensity and frequency will be assessed through a short questionnaire. 8) Potential moderators: Postpartum depression (PPD) will be assessed using the Edinburgh Postnatal Depression Scale (EPDS).^[[48]](#endnote-48)^ Women found to have PPD (defined as an EPDS score ≥12) will be assessed by a study clinician and offered a referral to services. They will be allowed to continue in the study. Any woman screening in for self-harm or harm to others will be immediately referred for mental health evaluation. We will assess appetite and sleep using real-time visual analog scales in the app. 9) Satisfaction: Likert scale for satisfaction and willingness to recommend to a friend.

| **Table 3: Measurements**  **Variable** | **Day 1, 2, or 3 pp** | **Visit 1**  **(6 wks pp)** | **Visit 2**  **(6 months pp)** | **Visit 3**  **(12 months pp)** |
| --- | --- | --- | --- | --- |
| Demographics Questionnaire (Based on Behavioral Risk Surveillance System (BRFSS) |  | X |  |  |
| Height |  | X |  |  |
| Medical History Update |  | X | X | X |
| Urine hcg* |  |  | X | X |
| **Clinical Outcomes** | | | | |
| Weight |  | X | X | X |
| Waist circumference |  | X | X | X |
| Heart Rate |  | X | X | X |
| Blood Pressure |  | X | X | X |
| Fasting lipid profile |  | X | X | X |
| HbA1c |  | X | X | X |
| TSH |  | X | X | X |
| OGTT (only for GDM women) |  | X |  |  |
| Fasting glucose |  | X | X | X |
| Fasting insulin |  | X | X | X |
| Adiponectin |  | X | X | X |
| hsCRP |  | X | X | X |
| **Behavioral Outcomes** | | | | |
| Adapted Pregnancy Physical Activity Questionnaire (PPAQ) |  | X | X | X |
| 2005 Block FFQ |  | X | X | X |
| Breastfeeding Questionnaire |  | X | X | X |
| **Mediating Variables** | | | | |
| Social Support for Eating |  | X | X | X |
| Social Support for Exercise |  | X | X | X |
| Self Efficacy Survey for Diet and Exercise Behaviors |  | X | X | X |
| Readiness to Change Questionnaire |  | X | X | X |
| Perceived Stress Scale |  | X | X | X |
| Edinburgh Postnatal Depression Scale (EPDS) |  | X | X | X |
| Sleep Questionnaire |  | X | X | X |
| Satisfaction |  | X | X | X |

*NOTE: Subjects will not take a urine hcg test at 6 weeks because the hcg would be expected to be elevated from pregnancy and would not be able to identify a new pregnancy. All subjects will take a pregnancy test at every subsequent study visit. In addition, if subject reports new amenorrhea, the subject will be asked to come in for a pregnancy test. If results indicate exclusionary condition such as pregnancy, subject will be removed from study and referred for appropriate clinical follow-up care. These subjects will be excluded as they would affect study data and because these conditions may require different care. If a subject is diagnosed with diabetes at any visit, we will ask their permission to continue to follow them. Permission will be requested to obtain medical records for the pregnancy to look at recurrence of pregnancy complications in both groups.

*NOTE: Measures will be taken to review subject data and diagnose diabetes according to American Diabetes Association (ADA) guidelines. The following values at the specified timepoints throughout the study year indicate that the subject will need to repeat the abnormal test to confirm the diagnosis of diabetes:

• At the 6-week visit, if the subject has a fasting glucose value >126 or a 2-hour OGTT glucose value >200. The HbA1c value collected at the 6-week visit is exploratory and is not clinically used to diagnose diabetes at 6-weeks, given that the result will be affected by hyperglycemia during pregnancy.

• At the 6-month and 12-month visits, if the subject has a fasting glucose value > 126 or a 2-hour OGTT glucose value >200 or if the HbA1c value is >7.0. For HbA1C values between 6.5 and 7.0, we will use the values from the OGTT as the standard for making a diagnosis for DM.

If two of the subject’s initial clinical tests exceed these values, they will be diagnosed with diabetes at that timepoint. If one of the subject’s initial clinical tests exceeds these values we will schedule a repeat fasting glucose, OGTT, or HbA1c. If the subject’s repeat test result exceeds these values, the subject will be diagnosed with diabetes. Subjects diagnosed with diabetes at the 6 week visit will be excluded from the study. If a subject is diagnosed with diabetes at any visit other than the 6 week visit, we will continue to follow them. This is to ensure that we can collect data on all randomized subjects, including body weight which is the primary outcome of the study. We will assist all subjects diagnosed with diabetes with a referral to an appropriate provider to manage their condition.

Validated Questionnaires

1. Demographics Questionnaire (Based on the Behavioral Risk Factor Surveillance System (BRFSS). http://hmcrc.srph.tamhsc.edu/Measures/BRFSS%20Demographic.pdf

2. Social Support for Eating Habits Survey - Sallis. http://www-rohan.sdsu.edu/faculty/sallis/socialsupport-eatinghabits.pdf.

3. Social Support for Physical Activity - Sallis. http://www-rohan.sdsu.edu/faculty/sallis/socialsupport-exercise.pdf.

4. Self Efficacy Survey for Diet and Exercise Behaviors. http://www-rohan.sdsu.edu/faculty/sallis/self-efficacy-coverandexercise.pdf

5. Perceived Stress Scale. http://www.macses.ucsf.edu/Research/Psychosocial/notebook/PSS10.html.

6. Edinburgh Post-Partum Depression Scale (EPDS)* http://www.fresno.ucsf.edu/pediatrics/downloads/edinburghscale.pdf.

7. 2005 Block FFQ

8. Pregnancy Physical Activity Questionnaire (PPAQ) http://www.ipaq.ki.se/.

9. Breastfeeding

10. Readiness to Change Questionnaire

http://www.rrh.org.au/publishedarticles/article_print_802.pdf

*NOTE: The Edinburgh Depression screen will be scored before the participant completes the study visit. A score ≥12 on the Edinburgh Depression Screen will prompt evaluation by a clinician, consistent with the clinical practice of the outpatient obstetric practices and the cutoffs published in the most current literature.^[[49]](#endnote-49)^ Per usual clinical care, any participant deemed acutely suicidal will be sent to the ED for emergent evaluation by psychiatry. Participants deemed to be at risk for depression, but not acutely suicidal, will be referred for further care.

For this study, these questionnaires will be organized according to topic into 4 groups and combined into one single computer administered study questionnaire to make them readable and user-friendly. To facilitate this grouping, the questionnaires have undergone some format changes and some questions have been removed.

**Measures for intervention group only**:

1) Behavioral tracking: Data from tracking through the *Fit After Baby* app will be analyzed to assess adherence to the program, including weekly weigh-ins (via provided scale), days with recorded physical activity data (transmitted by wearing Fitbit (provided), recorded real-time in the app, and/or manual entry), and days with dietary intake logged (via Fitbit app).

2) Engagement: We will collect data on 1) number of days using the app, 2) responses to prompts, 3) points and badges earned, and 4) number of interactions with lifestyle coach.

**Focus Groups:**

Subjects will be asked to attend a 1-2 hour focus groups via virtual zoom meeting. The groups will consist of up to 8 participants. There will be up to 8 sessions, each lasting no longer than 2 hours. They will be encouraged to respond to the facilitators’ questions and to each other’s comments on topics such as: barriers and facilitators to adoption and engagement of the Fit After Baby program. Participants will be recruited from the pool of participants who were involved in the FAB intervention group. Research staff will utilize a COMIRB approved focus group discussion guide to conduct the focus groups. Subjects will be allowed to take breaks as necessary. Participants will receive a $50 gift card as compensation for taking the time to come in person to provide us with their additional feedback. Group zoom sessions will be recorded and recordings will be stored on a secure server at the University of Colorado.

The patient re-consent process will be conducted using a REDCap-based electronic consent form. The consent form has been developed in REDCap, a secure, web-based, HIPAA-compliant, data collection platform with a user management system allowing project owners to grant and control varying levels of access to data collection instruments and data (e.g. read only, de-identified-only data views) for other users. Consent forms signed in RedCap will be converted to PDF and uploaded in the participants EMR per protocol.

Patient signatures will be obtained using a types signature. Upon completion of the consent, patients will be provided with a copy of their version of the consent document by allowing them to download a PDF of their consent form. The option to have the consent printed and mailed to participant by study staff will be available.

**Statistical Analysis**: The analysis for the primary outcomes will be intent-to-treat, using longitudinal mixed methods to account for loss to follow-up and censored participants. Due to the longitudinal nature of this study, we will assess the change in weight from the 6-week study visit to weight at 6 and 12 months by a mixed model analysis with an exchangeable correlation structure and adjustment for weight gain during pregnancy, and breastfeeding (a mediating effect). Within this model, we can further assess differences in weight between various time points by contrasts. We will also compare the delta weight prepregnancy to 6 and 12 months postpartum in the intervention group to the control group using a multivariate regression model, adjusting for age and race. Secondary clinical outcomes will be compared between groups by a generalized linear mixed model analysis, similar to the primary outcome analysis. When necessary, adjustment for multiple comparisons will be made. We will assess reach, effectiveness, and adoption per the RE-AIM model to prepare for a pragmatic trial (Table 2). We will use content-based analysis on focus group data to assess perceived barriers and facilitators to success of the intervention.

**Anticipated results**: Primary outcome: We expect that women who participate in the *Fit After Baby* program will demonstrate increased weight loss in the postpartum year vs. the Text4Baby control group. Secondary outcomes**:** We expect an improvement in cardiometabolic risk factors among women randomized to the Fit After Baby program. We anticipate that increased engagement and satisfaction will be associated with increased weight loss. We expect that decreased caloric intake relative to expenditure and increased physical activity will help to mediate weight loss. We anticipate that postpartum depression may interfere with weight loss as it will decrease engagement with the program, and appetite and sleep may also moderate weight loss.

| **Table 2: Relevant RE-AIM Outcomes** | **Measures/Data Source** | |
| --- | --- | --- |
| **REACH** | | |
| 1) Proportion of approached women agreeing to participate  2) Proportion of recruited women initiating program | | 1) Recruitment data  2) App usage data |
| **EFFECTIVENESS** | | |
| 1) Postpartum weight loss  2) Change in physical activity  3) Change in dietary intake | | 1) Change in weight from baseline and pre-pregnancy weight to 6 and 12 months postpartum  2) Change physical activity assessed by modified PPAQ  3) Change in diet assessed by 24-hour recall |
| **ADOPTION** | | |
| 1) Engagement with app and lifestyle coach  2) Acceptability and perceived barriers to success of intervention | | 1) Usage data from app (daily use, weight, diet, physical activity tracking, responses to prompts, points earned); number of interactions with lifestyle coach  2) Satisfaction; Participant focus group data |

6. RISKS AND DISCOMFORTS

Previous experience, the non-invasive nature of the proposed study, and the close supervision of subjects at the study site will minimize the potential risks. The co-investigators have extensive experience performing the study procedures. Therefore, the anticipated risk to the subjects from these studies is minimal.

1. Oral glucose tolerance test: The glucose solution has a sweet taste that may be considered unpleasant and may be associated with nausea or, rarely, vomiting. Subjects will be monitored by trained personnel during the OGTT.

2. Blood collection: Blood draws may cause fainting, tenderness, hematoma formation, and rarely, infection. Sterilization of the skin and collection of blood by skilled nurses minimizes these risks.

3. Questionnaires: It may be tiring to fill out questionnaires, but the subject will be given as much time as needed to complete them. Also, a participant may feel uncomfortable answering questions of a personal nature, but subject can always choose not to answer any question.

4. Weight Loss: Weight loss may occur too rapidly resulting in weakness and fatigue; however, weight loss will be gradual and overseen by the lifestyle coach, a dietitian with training to minimize this risk.

5. Confidentiality: The Principal Investigator and her staff will take all reasonable measures to protect the confidentiality of each subject’s records and data. Subject data will be referenced by number only. Research data kept online will be kept secure and confidential through extensive password protection, username anonymity, and redirected emails. Data will be stored in RedCap.

c. Device complications/malfunctions: none

d. Psychosocial (non-medical) risks: none anticipated

e. Financial Risks: none

6.1 POTENTIAL BENEFITS

a. Potential benefits to participating individuals

No direct individual benefit is likely to come from participating in this study. This study will explore the hypothesis that a novel lifestyle intervention program modeled upon the DPP for overweight/obese postpartum women at elevated cardiometabolic risk can successfully affect healthy diet and physical activity habits leading to weight loss and possibly reduce the risk for future development of type 2 diabetes and cardiovascular disease in the study population. We believe that the importance of gaining this knowledge outweighs the minimal risk posed to the subjects. Some women in both groups may receive a diagnosis of type 2 diabetes during the study. They may consequently be able to receive earlier care for this condition than they would have otherwise.

b. Potential benefits to society

The successes and failures of this program will help identify the unique obstacles that enable and prevent this at-risk population from reducing their type 2 diabetes and cardiovascular disease risk through diet and physical activity regimens. Lifestyle intervention programs like the DPP remain an extremely effective treatment for reducing cardiometabolic risk in at-risk populations; however, they are time- and labor-intensive, making them difficult to translate/replicate in at-risk populations with unique time restrictions (like new mothers/mothers of very young children/women in the first year postpartum). Identifying alternative lifestyle programs that are effective in this population may be therapeutically useful in this large segment of the population at elevated risk.

Protocol Management: The studies will be conducted in the well-supervised CCTSI at the University of Colorado. The subjects will be under constant observation by skilled professionals highly qualified to perform the study procedures. Procedures will be terminated immediately if potentially serious side effects develop. In the event of adverse reactions, medical care will be immediately available.

Data Management: The original recording of an observation will be retained as the source document. The principal investigator will prepare and maintain complete and accurate study documentation in compliance with good clinical practice standards and applicable federal, state, and local laws, rules, and regulations. Study documentation shall be made available at the investigator’s site upon request for inspection, copying, review and audit at reasonable times by any regulatory agency. The investigator will promptly take any reasonable steps that are requested as a result of an audit to correct deficiencies in the study documentation. Data confidentiality will be maintained and regulations regarding access to data by those other than the study staff will be followed. All information will be entered into the study dataset by coded entry only. The code identification data will be kept in a locked file available only to the principal investigator unless requested by the appropriate regulatory authority. Local data will be stored and collected using RedCap.

Study data will be collected and managed using REDCap (Research Electronic Data Capture). REDCap is a secure web application designed to support data capture for research studies, providing user-friendly web-based case report forms, real-time data entry validation (e.g. for data types and range checks), audit trails and a de-identified data export mechanism to common statistical packages (SPSS, SAS, Stata, R/S-Plus). The system was developed by a multi-institutional consortium which includes University of Colorado–Denver and was initiated at Vanderbilt University. The database is hosted at the University of Colorado–Denver Development and Informatics Service Center (DISC), which will be used as a central location for data processing and management. REDCap data collection projects rely on a thorough study-specific data dictionary defined in an iterative self-documenting process by all members of the research team with planning assistance from the DISC. This iterative development and testing process results in a well-planned data collection strategy for individual studies. REDCap also includes a powerful tool for building and managing online surveys. The research team can create and design surveys in a web browser and engage potential respondents using a variety of notification methods. REDCap is flexible enough to be used for a variety of types of research and provides an intuitive user interface for database and survey design and data entry. ^[[50]](#endnote-50)^

Progress Reports: Annual progress reports will be submitted to the COMIRB describing all of the study activities.

a. Independent monitoring of source data: none

b. Safety monitoring: A physician reviews all laboratory results as they become available. A physician involved with the study is available by pager 24 hours/day seven days/week if any difficulties arise.

c. Outcomes monitoring: The Principal Investigator Dr. Jacinda Nicklas, will be responsible for reviewing and reporting all data and safety data within 24 hours of receipt. We will address abnormal lab values as follows:

1) Glucose, HbA1c: We will review subject data in accordance with the American Diabetes Association (ADA) guidelines for the diagnosis of diabetes. The following values at the specified timepoints throughout the study year indicate that the subject will need to repeat the abnormal test to confirm the diagnosis of diabetes:

For participants with a recent history of GDM who complete an oral glucose tolerance test: At the 6-week visit, if the subject has a fasting glucose value >126 or a 2-hour OGTT glucose value >200. The HbA1c value collected at the 6-week visit is exploratory and is not clinically used to diagnose diabetes at 6-weeks, given that the result will be affected by hyperglycemia during pregnancy.

For all other participants (who will not complete an OGTT):
At the 6 week visit, if the subject has a fasting glucose value > 126.

• At the 6-month and 12-month visits, if the subject has a fasting glucose value > 126 or if the HbA1c value is ≥6.5.

If two of the subject’s initial clinical tests exceed these values, they will be diagnosed with diabetes at that timepoint. If one of the subject’s initial clinical tests exceeds these values we will schedule a repeat fasting glucose, OGTT, or HbA1c. If the subject’s repeat test result exceeds these values, the subject will be diagnosed with diabetes. Subjects diagnosed with diabetes at the 6 week visit will be excluded from the study. If a subject is diagnosed with diabetes at any visit other than the 6 week visit, we will continue to follow them. This is to ensure that we can collect data on all randomized subjects, including body weight which is the primary outcome of the study. We will assist all subjects diagnosed with diabetes with a referral to an appropriate provider to manage their condition.

2. Fasting lipid profile:

Based on the 2013 ACC/AHA blood cholesterol guideline statins should be prescribed in patients without clinical atherosclerotic cardiovascular disease with a LDL-C above or equal to 190 mg/dL, if a subject has a LDL-C above 190mg/dL, the study physician will call the participant to let her know that her cholesterol level was elevated and that she should contact her PCP. If a subject has a fasting TG level above 800 mg/dL, the study physicianwill call the participant to let her know that her triglyceride level was elevated and that she should contact her PCP.

3. TSH: If a participant has a TSH out of the normal range at any timepoint she will be contacted and advised to contact her PCP.

d. Adverse event reporting guidelines: The principal investigator is responsible for reporting all adverse events according to guidelines. Expected and unexpected adverse events associated with this protocol will be reported to COMIRB per guidelines. All potential adverse events will be reviewed by the study staff at our weekly meeting.

7. DATA SAFETY AND SECURITY

A number of measures will be undertaken to ensure that participants’ confidentiality is maintained and that data are secure. These include:

1. Study activities involving human subjects will not begin until approval has been granted from COMIRB.
2. Recruitment and screening data, data from focus groups, discussion threads, and survey data will be stored on a secure server at the University of Colorado.
3. Survey Data Storage: Surveys will be housed on Research Electronic Data Capture (REDCap), a secure site for online data collection that is HIPAA compliant, and links to the surveys sent to participant e-mails.
4. All data collected from participants will be identified by ID number only, and will be encrypted when transmitted from the Internet. At the end of the research study, all data are permanently de-identified for archive, according to the schedule established by COMIRB.
5. All investigators and staff have completed the CITI Basic Course and CITI Health Information Privacy and Security (HIPS) Course.
6. Data collected through the Fit After Baby application and website will be protected via security measures. The dataset will include ID number, email address, and cell phone number (to enable emailing and texting functions), but no other personally identifying information. The security measures include monitoring data, protecting privacy of subjects, and maintaining confidentiality of data. The Data Center (managed by Google, Inc.) has a Global Compliance function that is responsible for legal and regulatory compliance as well as a Global Internal Audit function responsible for reviewing and auditing adherence to compliance requirements, including Sarbanes-Oxley and Payment Card Industry standards (PCI). The security measures include physical measures, access protection, storage protection, network security, measures to protect data transfer, measures to protect against data loss and security breaches, and measures to address disaster recovery and continuity of services.

1) **Physical Security Measures:**

Staff at headquarters are responsible for the physical security of Comprendo’s offices. Google staff and data center facilities are protected with Google’s security officers who are qualified with training to protect high security enterprises with mission-critical infrastructures. The servers feature a Hardware Firewall and run active intrusion monitoring. All access to web portal and any client data is secured over HTTPS using SSL 256-bit encryption. Additionally, it is policy for all staff members with access to participant data to receive training as a HIPAA Privacy Associate, and be trained in HPIAA Privacy Rights.

Google’s data centers are geographically distributed and employ a variety of physical security measures. The standard physical security controls implemented at each Google data center are composed of well-known technologies and follow generally accepted industry best practices: custom designed electronic card access control systems, alarm systems, interior and exterior cameras, and security guards. Access to areas where systems, or system components, are installed or stored are segregated from general office and public areas such as lobbies. The cameras and alarms for each of these areas are centrally monitored for suspicious activity, and the facilities are routinely patrolled by security guards. Google’s facilities use high resolution cameras with video analytics and other systems to detect and track intruders. Activity records and camera footage are kept for later review, should it become necessary. Access to all data center facilities is restricted to authorized Google employees, approved visitors, and approved third parties whose job it is to operate the data center. Google maintains a visitor access policy and set of procedures stating that data center managers must approve any visitors in advance for the specific internal areas they wish to visit. The visitor policy also applies to Google employees who do not normally have access to data center facilities. Google audits who has access to its data centers on a quarterly basis to help ensure that only appropriate personnel have access to each floor. Google restricts access to its data centers based on role, not position. As a result, even most senior executives at Google do not have access to Google data centers.

**2) Access:** All account data are secured by standard https security protocols. Maintenance access is secured by Google Admin Account access and controlled by the Chief Technology Officer of the organization through the use of Web Admin tools. Approval to use these Web Admin tools is provided by the CTO to a limited set of employees based on job responsibilities and position within the organization. A separate development and testing environment will be used to help minimize access to participant data.

**3) Storage:** The architecture is built on the Google Cloud platform and takes advantage of Google's strong security measures and data centers. (<https://cloud.google.com/files/Google-CommonSecurity-WhitePaper-v1.4.pdf>)

**4) Network security:** Google employs multiple layers of defense to help protect the network perimeter from external attacks. Only authorized services and protocols that meet Google’s security requirements are permitted to traverse the company’s network. Unauthorized packets are automatically dropped.

**5) Transport security**: Google provides many services that make use of the Hypertext Transfer Protocol Secure (HTTPS) for secure browser connections. Information sent via HTTPS is encrypted from the time it leaves Google until it is received by the recipient’s computer.

**6) Data Transfer security:** Electronic transmission of Personal Health Information (PHI) or Personally Identifiable Information (PII) over the internet (including by email), file transfers or other data transfer modalities, will be encrypted. The account data is secured by standard https secure protocol (Secure Socket Layer - SSL). No data containing PHI or PII may be sent from or forwarded to an external account such as Gmail, Yahoo mail, etc.

**7) Data Loss/Security Breach:** Examples of security breaches include (1) lost or stolen desktops laptops, USB drives, CD/DVD/Zip Drives, etc. with stored data; (2) a compromised account which is used to look up data (e.g., unauthorized user has had access to the account); (3) a compromised work station or server that contains data; and (4) accidental disclosure or data to unauthorized recipients (e.g., sending data to an incorrect email address). In compliance with HIPAA, it is policy to notify participants alerting them to breaches “without unreasonable delay,” but no later than 60 days after discovery of the breach. Notice to the secretary of Health and Human Services (HHS) and prominent media outlets about breaches involving more than 500 patients.

**8) Disaster Recovery and Continuity of Services:** To minimize service interruption due to hardware failure, natural disaster, or other catastrophe, Google implements a disaster recovery program at all of its data centers. This program includes multiple components to minimize the risk of any single point of failure, including the following: 1) Data replication and backup: application data is replicated to multiple systems within a data center, and in some cases also replicated to multiple data centers. 2) Google operates a geographically distributed set of data centers that is designed to maintain service continuity in the event of a disaster or other incident in a single region. High-speed connections between the data centers help to support swift failover. Management of the data centers is also distributed to provide location-independent, around-the-clock coverage, and system administration. 3) In addition to the redundancy of data and regionally disparate data centers, Comprendo also has a business continuity plan for its headquarters in Santa Barbara, CA. This plan accounts for major disasters, such as a seismic event or a public health crisis, and it assumes people and services may be unavailable for up to 10 days. This plan is designed to enable continued operations of services for customers. Comprendo destroys PHI or PII on request by individuals or covered entities according to HIPAA policies except in exceptional circumstances. Where destruction is not possible the data will be anonymized to the extent possible while maintaining the integrity of the platform, and access to the data will be restricted.

**9) Protection of data from third-party websites and devices:**

Participants will need to establish accounts with Fitbit for diet and activity monitoring. They will establish these accounts with an email address and a password. They will have the option of inputting personal information, (i.e. name, gender, height, weight, birthday, and time zone). These data are subject to the privacy policies detailed on these third party websites. Personal data collected on these websites will not be transmitted to Comprendo or University of Colorado. Data collected through Fitbit will be transferred to identified only by email address, and used to provide individualized feedback to the users.

1. Mosca L, Benjamin EJ, Berra K, Bezanson JL, Dolor RJ, Lloyd-Jones DM, Newby LK, Pina IL, Roger VL, Shaw LJ, Zhao D, Beckie TM, Bushnell C, D’Armiento J, Kris-Etherton PM, Fang J, Ganiats TG, Gomes AS, Gracia CR, Haan CK, Jackson EA, Judelson DR, Kelepouris E, Lavie CJ, Moore A, Nussmeier NA, Ofili E, Oparil S, Ouyang P, Pinn VW, Sherif K, Smith SC, Sopko G, Chandra-Strobos N, Urbina EM,Vaccarino V, Wenger NK. Effectiveness-based guidelines for the prevention of cardiovascular disease in women–2011 update: a guideline from the American Heart Association. Circulation. 123:1243–1262, 2011. [↑](#endnote-ref-1)
2. Rich-Edwards JW, Fraser A, Lawlor DA, Catov JM. Pregnancy characteristics and women’s future cardiovascular health: An underused opportunity to improve women’s health? Epidemiol Rev. 2014;36(1):57-70. PubMed PMID: 24025350. [↑](#endnote-ref-2)
3. Gunderson EP, Chiang V, Pletcher MJ, Jacobs DR, Quesenberry CP, Sidney S, Lewis CE. History of gestational diabetes mellitus and future risk of atherosclerosis in mid-life: the Coronary Artery Risk Development in Young Adults study. J Am Heart Assoc. 2014 Mar 12;3(2):e000490. [↑](#endnote-ref-3)
4. Tobias DK, Hu FB, Forman JP, Chavarro J, Zhang C. Increased risk of hypertension after gestational diabetes mellitus: findings from a large prospective cohort study. Diabetes Care. 2011 Jul;34(7):1582-4. [↑](#endnote-ref-4)
5. Rich-Edwards JW. The predictive pregnancy: What complicated pregnancies tell us about a mother’s future cardiovascular risk. Circulation. 125:1136-1338, 2012. PubMed PMID: 22354939. [↑](#endnote-ref-5)
6. Fraser A, Nelson SM, MacDonald-Wallis C, Cherry L, Butler E, Sattar N, Lawlor DA. Associations of pregnancy complications with calculated cardiovascular disease risk and cardiovascular risk factors in middle age: the Avon Longitudinal Study of Parents and Children. Circulation 2012;125:1367-80. PMID: 22344039. [↑](#endnote-ref-6)
7. Fraser A, Nelson SM, MacDonald-Wallis C, Cherry L, Butler E, Sattar N, Lawlor DA. Associations of pregnancy complications with calculated cardiovascular disease risk and cardiovascular risk factors in middle age: the Avon Longitudinal Study of Parents and Children. Circulation 2012;125:1367-80. PMID: 22344039. [↑](#endnote-ref-7)
8. Rich-Edwards JW. The predictive pregnancy: What complicated pregnancies tell us about a mother’s future cardiovascular risk. Circulation. 125:1136-1338, 2012. PubMed PMID: 22354939 [↑](#endnote-ref-8)
9. http://www.cdc.gov/women/lcod/2013/index.htm (accessed October 5, 2015) [↑](#endnote-ref-9)
10. Mosca L, Benjamin EJ, Berra K, et al. Effectiveness-based guidelines for the prevention of cardiovascular disease in women–2011update: a guideline from the American heart association. J Am Coll Cardiol. 2011;57:1404–23 [↑](#endnote-ref-10)
11. Must A, Spadano J, Coakley EH, Field AE, Colditz G, Dietz WH. The disease burden associated with overweight and obesity. JAMA. 1999 Oct 27;282(16):1523-9. PubMed PMID: 10546691. [↑](#endnote-ref-11)
12. Ogden CL, Carroll MD, Flegal KM. Prevalence of obesity in the United States. JAMA. 2014 Jul;312(2):189-90. [↑](#endnote-ref-12)
13. Finkelstein, EA, Trogdon, JG, Cohen, JW, and Dietz, W. Annual medical spending attributable to obesity: Payer- and service-specific estimates. Health Affairs. 28(5): w822-w831, 2009. [↑](#endnote-ref-13)
14. Thompson D, Edelsberg J, Colditz GA, Bird AP, Oster G. Lifetime health and economic consequences of obesity. Arch Int Med. 159(18): 2177-2183, 1999. [↑](#endnote-ref-14)
15. Artal R, Lockwood CJ, Brown HL. Weight gain recommendations in pregnancy and the obesity epidemic. Obstet Gynecol. 115(1):152-5, 2010. [↑](#endnote-ref-15)
16. Villamor E, Cnattingius S. Interpregnancy weight change and risk of adverse pregnancy outcomes: a population-based study. Lancet. 368(9542):1164-70, 2006. [↑](#endnote-ref-16)
17. Bentley-Lewis, R., Levkoff, S., Stuebe, A., & Seely, E. W. Gestational diabetes mellitus: Postpartum opportunities for the diagnosis and prevention of type 2 diabetes mellitus. Nature Clinical Practice Endocrinology & Metabolism, 4(10), 552-558, 2008. [↑](#endnote-ref-17)
18. Smith GN. The development of preeclampsia provides a window of opportunity for early cardiovascular risk screening and intervention. Expert Rev Obstet and Gynecol. 2009;4:355-7. [↑](#endnote-ref-18)
19. Knowler WC, Barrett-Connor E, Fowler SE, Hamman RF, Lachin JM, Walker EA, Nathan DM; Diabetes Prevention Program Research Group. Reduction in the incidence of type 2 diabetes with lifestyle intervention or metformin. N Engl J Med. 2002 Feb 7;346(6):393-403. [↑](#endnote-ref-19)
20. Eckel RH, Jakicic JM, Ard JD, de Jesus JM, Houston Miller N, Hubbard VS, Lee IM, Lichtenstein AH, Loria CM, Millen BE, Nonas CA, Sacks FM, Smith SC Jr, Svetkey LP, Wadden TA, Yanovski SZ, Kendall KA, Morgan LC, Trisolini MG, Velasco G, Wnek J, Anderson JL, Halperin JL, Albert NM, Bozkurt B, Brindis RG, Curtis LH, DeMets D, Hochman JS, Kovacs RJ, Ohman EM, Pressler SJ, Sellke FW, Shen WK, Smith SC Jr, Tomaselli GF; American College of Cardiology/American Heart Association Task Force on Practice Guidelines. 2013 AHA/ACC guideline on lifestyle management to reduce cardiovascular risk: a report of the American College of Cardiology/American Heart Association Task Force on Practice Guidelines. Circulation. 2014 Jun 24;129(25 Suppl 2):S76-99. doi: 10.1161 /01.cir.0000437740.48606.d1. Epub 2013 Nov 12. [↑](#endnote-ref-20)
21. Bao W, Tobias DK, Bowers K, Chavarro J, Vaag A, Grunnet LG, Strøm M, Mills J, Liu A, Kiely M, Zhang C. Physical activity and sedentary behaviors associated with risk of progression from gestational diabetes mellitus to type 2 diabetes mellitus: a prospective cohort study. JAMA Intern Med. 2014 Jul;174(7):1047-55. [↑](#endnote-ref-21)
22. Hedderson M, Ferrara A. A call to increase physical activity among women of reproductive age: is it possible? JAMA Intern Med. 2014 Jul;174(7):1056-7. [↑](#endnote-ref-22)
23. Huang Z, Willett WC, Manson JE, Rosner B, Stampfer MJ, Speizer FE, Colditz GA. Body weight, weight change, and risk for hypertension in women. Ann Intern Med. 1998 Jan 15;128(2):81-8. PubMed PMID: 9441586. [↑](#endnote-ref-23)
24. Catalano PM, Ehrenberg HM. The short- and long-term implications of maternal obesity on the mother and her offspring. BJOG. Oct;113(10):1126-33, 2006. [↑](#endnote-ref-24)
25. Glazer NL, Hendrickson AF, Schellenbaum GD, Mueller BA. Weight change and the risk of gestational diabetes in obese women. Epidemiology. 15(6):733, 2004. [↑](#endnote-ref-25)
26. Hoedjes M, Berks D, Vogel I, Franx A, Duvekot JJ, Oenema A, Steegers EA, Raat H. Motivators and barriers to a healthy postpartum lifestyle in women at increased cardiovascular and metabolic risk: a focus-group study. Hypertens Pregnancy. 2012;31(1):147-55. doi: 10.3109/10641955.2010.544803. Epub 2011 Jan20. PubMed PMID: 21250888. [↑](#endnote-ref-26)
27. Hoedjes M, Berks D, Vogel I, Franx A, Visser W, Duvekot JJ, Habbema JD, Steegers EA, Raat H. Effect of postpartum lifestyle interventions on weight loss, smoking cessation, and prevention of smoking relapse: a systematic review. Obstet Gynecol Surv. 2010 Oct;65(10):631-52. doi: 10.1097/OGX.0b013e3182077f64. Review. PubMed PMID: 21182803. [↑](#endnote-ref-27)
28. Ferrara A, Hedderson MM, Albright CL, Ehrlich SF, Quesenberry CP Jr, Peng T, Feng J, Ching J, Crites Y. A pregnancy and postpartum lifestyle intervention in women with gestational diabetes mellitus reduces diabetes risk factors: a feasibility randomized control trial. Diabetes Care. 2011 Jul;34(7):1519-25. [↑](#endnote-ref-28)
29. Ratner RE, Christophi CA, Metzger BE, Dabelea D, Bennett PH, Pi-Sunyer X, Fowler S, Kahn SE; Diabetes Prevention Program Research Group. Prevention of diabetes in women with a history of gestational diabetes: effects of metformin and lifestyle interventions. J Clin Endocrinol Metab. Dec;93(12):4774-9, 2008. [↑](#endnote-ref-29)
30. Østbye T, Krause KM, Lovelady CA, et al. Active Mothers Postpartum (AMP): A randomized controlled weight loss intervention trial. Am J Prev Med. 37:173–180, 2009. [↑](#endnote-ref-30)
31. Sarwer DB, Allison KC, Gibbons LM, Markowitz JT, Nelson DB. Pregnancy and Obesity: A review and agenda for future research. J Women’s Health.15(6):720, 2006. [↑](#endnote-ref-31)
32. Nicklas JM and Barbour LA. Optimizing Weight for Maternal and Infant Health – Tenable, or Too Late? Expert Review of Endocrinology & Metabolism. 1–16, 2014. [↑](#endnote-ref-32)
33. Nicklas JM, Seely EW, Zera CA, Abdul-Rahim ZS, Rudloff ND, Levkoff SE. Identifying novel approaches to preventing type 2 diabetes in women with a history of gestational diabetes. BMC Pregnancy and Childbirth. 11:23, 2011. PubMed PMID: 25837258. [↑](#endnote-ref-33)
34. Dasgupta K, Da Costa D, Pillay S, De Civita M, Gougeon R, Leong A, Bacon S, Stotland S, Chetty VT, Garfield N, Majdan A, Meltzer S. Strategies to optimize participation in diabetes prevention programs following gestational diabetes: A focus group study. PLOS ONE. 8(7): 2013. [↑](#endnote-ref-34)
35. Cusimano MC, Pudwell J, Roddy M, Cho CK, Smith GN. The maternal health clinic: an initiative for cardiovascular risk identification in women with pregnancy-related complications. Am J Obstet Gynecol. 2014 May;210(5):438.e1-9. doi: 10.1016/j.ajog.2013.12.001. Epub 2013 Dec 4. PubMed PMID: 24316270. [↑](#endnote-ref-35)
36. Menke A, Casagrande S, Geiss L, Cowie CC. Prevalence of and Trends in Diabetes Among Adults in the United States, 1988-2012. JAMA. 2015 Sep 8;314(10):1021-9. doi: 10.1001/jama.2015.10029. PubMed PMID: 26348752. [↑](#endnote-ref-36)
37. Breton ER, Fuemmeler BF, Abroms LC. Weight loss – there is an app for that! But does it adhere to evidence-informed practices? Trans Behav Med 2011 December 1(4) 523-529. [↑](#endnote-ref-37)
38. Khaylis A, Yiaslas T, Bergstrom J, Gore-Felton C. A review of efficacious technology-based weight-loss interventions: five key components. Telemed J E Health. 2010 Nov;16(9):931-8. doi: 10.1089/tmj.2010.0065. Review. PubMed PMID: 21091286; PubMed Central PMCID: PMC3000900. [↑](#endnote-ref-38)
39. Azar KM, Lesser LI, Laing BY, Stephens J, Aurora MS, Burke LE, Palaniappan LP. Mobile applications for weight management: theory-based content analysis. Am J Prev Med. 2013 Nov;45(5):583-9. doi: 10.1016/j.amepre.2013.07.005. PubMed PMID: 24139771. [↑](#endnote-ref-39)
40. Conroy DE, Yang CH, Maher JP. Behavior change techniques in top-ranked mobile apps for physical activity. Am J Prev Med. 2014 Jun;46(6):649-52. doi: 10.1016/j.amepre.2014.01.010. PubMed PMID: 24842742. [↑](#endnote-ref-40)
41. Burke LE, Ma J, Azar KM, Bennett GG, Peterson ED, Zheng Y, Riley W, Stephens J, Shah SH, Suffoletto B, Turan TN, Spring B, Steinberger J, Quinn CC; American Heart Association Publications Committee of the Council on Epidemiology and Prevention, Behavior Change Committee of the Council on Cardiometabolic Health, Council on Cardiovascular and Stroke Nursing, Council on Functional Genomics and Translational Biology, Council on Quality of Care and Outcomes Research, and Stroke Council. Current Science on Consumer Use of Mobile Health for Cardiovascular Disease Prevention: A Scientific Statement From the American Heart Association. Circulation. 2015 Sep 22;132(12):1157-213. doi: 10.1161/CIR.0000000000000232. Epub 2015 Aug 13. PubMed PMID: 26271892. [↑](#endnote-ref-41)
42. Smirnakis KV, Chasan-Taber L, Wolf M, Markenson G, Ecker JL, Thadhani R. Postpartum diabetes screening in women with a history of gestational diabetes. Obstet Gynecol. 2005 Dec;106(6):1297-303. PubMed PMID: 16319255. [↑](#endnote-ref-42)
43. Whittaker R, Matoff-Stepp S, Meehan J, Kendrick J, Jordan E, Stange P, Cash A, Meyer P, Baitty J, Johnson P, Ratzan S, Rhee K. Text4baby: development and implementation of a national text messaging health information service. Am J Public Health. 2012 Dec;102(12):2207-13. doi: 10.2105/AJPH.2012.300736. Epub 2012 Oct 18. PubMed PMID: 23078509; PubMed Central PMCID: PMC3519339. [↑](#endnote-ref-43)
44. Ratner RE, Christophi CA, Metzger BE, Dabelea D, Bennett PH, Pi-Sunyer X, Fowler S, Kahn SE; Diabetes Prevention Program Research Group. Prevention of diabetes in women with a history of gestational diabetes: effects of metformin and lifestyle interventions. *J Clin Endocrinol Metab*. Dec;93(12):4774-9, 2008. [↑](#endnote-ref-44)
45. Oken E, Taveras EM, Kleinman KP, Rich-Edwards JW, Gillman MW 2007 Gestational weight gain and child adiposity at age 3 years. Am J Obstet Gynecol 196(4):322.e1-8. [↑](#endnote-ref-45)
46. CDC. National Health and Nutrition Examination Survey (NHANES) Anthropometry Procedures Manual. CDC. January 2007. 45-46. [↑](#endnote-ref-46)
47. Chasan-Taber L, Schmidt MD, Roberts DE, Hosmer D, Markenson G, Freedson PS. Development and validation of a Pregnancy Physical Activity Questionnaire. Med Sci Sports Exerc. 2004 Oct;36(10):1750-60.PubMed PMID: 15595297. [↑](#endnote-ref-47)
48. Cox JL, Holden JM, Sagovsky R. (1987). Detection of postnatal depression. Development of the 10-item Edinburgh Postnatal Depression Scale. Br J Psychiatry. 150:782-6. [↑](#endnote-ref-48)
49. Venkatesh KK, Nadel H, Blewett D, Freeman MP, Kaimal AJ, Riley LE. Implementation of universal screening for depression during pregnancy: Feasibility and impact on obstetric care. Am J Obstet. Gynecol (2016) doi: 10.1016/j.ajog.2016.05.024 [↑](#endnote-ref-49)
50. P.A. Harris, R. Thielke, R. Taylor, J. Payne, N. Gonzalez, J.G. Conde. Research Electronic Data Capture (REDCap) - A metadata-driven methodology and workflow process for providing translational research informatics support. Journal of Biomedical Informatics, 2008 [↑](#endnote-ref-50)
