# Supplementary material for: The Fit After Baby randomized controlled trial: An mHealth postpartum lifestyle intervention for women with elevated cardiometabolic risk
Source: PLoS One. 2024 Jan 9;19(1):e0296244. doi: 10.1371/journal.pone.0296244 (PMC10775990; doi:10.1371/journal.pone.0296244)
Supplement: Attachment — Submitted filename: response to reviewers v2.docx [file pone.0296244.s004.docx]

Dear reviewers,

Thank you for your help improving the manuscript thus far. Below please find our responses to the latest round of comments.

Reviewer #4: Thank you very much for all the efforts to reply to my previous comments. Some clarifications are still required.

1. You state that "We also noted that the reference you provided referred to models for longitudinal count data and therefore would not apply to our data." This is far from being true. Your model casts into the generalized linear mixed framework, as well as the provided reference. It is well-known that the initial conditions may strongly bias the estimated coefficients, see also the Heckman model. Accordingly, the model must be modified accordingly and results compared with those presented in the current version of the paper.

Thank you for your comment; that is an excellent point. We have re-run all of our models including baseline values as a covariate. This led to some changes in our results and we have rewritten our manuscript accordingly. We have also rewritten the Methods to state that we have run our models with baseline as a covariates, and we have added this as a footnote to all tables.

2. According to your reply about the missing data mechanism is at random. This assumption is hardly tenable. Moreover, the nature of missing data mechanism does not depend on the amount of missingnes, please refer to the statistical literature on the topic and to Little's work in particular.

Thank you for the opportunity to clarify. We did not mean to imply that the nature of the missing data mechanism depended on the amount of missing data, but rather that because the amount of missing data was relatively low, it would be less likely to impact our conclusions even if the assumption of missing completely at random or missing at random was violated.

The table below provides a comparison of the baseline characteristics and randomization assignments of participants who did and did not have missing data. These two groups are quite similar, further supporting the idea that missing data in this study was unlikely to cause significant bias.

| **Baseline Characteristics** | **Missing data** | |  |
| --- | --- | --- | --- |
|  | **Yes** | **No** | **P values** |
| **Age** | 29.4 (5.4) | 31.3 (5.4) | 0.1639 |
| **BMI** | 32.9 (4.9) | 32.1 (5.0) | 0.5241 |
| **Weight(Kg)** | 89.0 (15.4) | 86.8 (14.8) | 0.5369 |
| **Group** |  |  | 0.8795 |
| Intervention | 16 (66.7%) | 37 (64.9%) |  |
| Control | 8 (33.3%) | 20 (35.1%) |  |
| **Affected by COVID** |  |  | 0.4974 |
| Yes | 5 (20.8%) | 16 (28.1%) |  |
| No | 19 (79.2%) | 41 (71.9%) |  |

Finally, because this is an RCT, conditioning on the baseline value of the outcome reduces the potential for bias (PMID: **22262640**). We have added a sentence about this comparison to the Results section and a sentence to the limitations.

3. Please, provide more details on the check of model's assumptions. It is rather unclear why the BLUP graph should provide information about the random effects distribution. Similarly, having a sample of 80 or more observations does not guarantee that normality is met. Even one outlying observation only may strongly affect the results.

Thank you for the opportunity to clarify. The BLUPs are an estimate of the random effects. Please see the following reference:

Christian Ritz. (2004). Goodness-of-Fit Tests for Mixed Models. *Scandinavian Journal of Statistics*, *31*(3), 443–458. http://www.jstor.org/stable/4616841
